# Near-Complete Genome Sequence of an H5N1 Avian Influenza Virus Strain Isolated from a Swan in Southwest Kazakhstan in 2006

**DOI:** 10.1128/MRA.00016-20

**Published:** 2020-03-26

**Authors:** Yerbol Burashev, Vitaliy Strochkov, Kulyaisan Sultankulova, Mukhit Orynbayev, Markhabat Kassenov, Nurlan Kozhabergenov, Kamshat Shorayeva, Sandugash Sadikaliyeva, Aisha Issabek, Meirym Almezhanova, Aziz Nakhanov, Irina Savitskaya, Kunsulu Zakarya

**Affiliations:** a Research Institute for Biological Safety Problems (RIBSP), Gvardeyskiy, Kazakhstan; b Faculty of Biology and Biotechnology, Al-Farabi Kazakh National University, Almaty, Kazakhstan; Portland State University

## Abstract

We report the near-complete genome sequence of an influenza H5N1 virus strain isolated from a dead swan on the southeastern Caspian seashore in 2006. The results of the surface protein HA phylogenetic analysis showed that the A/swan/Mangystau/3/2006 virus belongs to the EA-nonGsGD clade.

## ANNOUNCEMENT

Avian influenza virus (AIV), like the other influenza A viruses, belongs to the *Orthomyxoviridae* family, members of which contain eight segmented RNA molecules of negative polarity (1). Until spring 2005, the highly pathogenic avian influenza H5N1 (HPAI-H5N1) virus circulated only in the territory of East and Southeast Asia (2). However, in May and June of 2005, over 6,000 water birds of 8 wild species were found dead on Quinghai Lake in central China, and HPAI-H5N1 was detected in 15 birds of 6 wild species (3).

In the spring of 2005, like in the Chinese outbreaks of the disease caused by HPAI-H5N1, an outbreak occurred in Kazakhstan during the seasonal migration. In the northern regions of the country, during a short period, infection and death of domestic birds were reported. Analysis of the surface proteins detected molecular pathogenic markers typical for the Quinghai genotype of HPAI-H5N1 virus (4). The intravenous pathogenicity index of the isolated virus strains ranged from 2.34 to 2.69 (5).

Twelve samples were taken from trachea, lung, intestine, and brain of three dead swans found in Mangystau Oblast, Kazakhstan, during the outbreak. A single positive sample was introduced into an embryonated chicken egg. The virus strain A/swan/Mangystau/3/2006 (H5N1; SW/3/2006) was isolated from 10-day-old embryonated chicken eggs. The viral RNA was extracted from allantoic fluid using the RNeasy minikit (Qiagen, Germany) according to the manufacturer’s instructions. All eight gene segments were amplified in the SuperScript one-step reverse transcriptase PCR (RT-PCR) system with Platinum Taq DNA polymerase (Invitrogen SRL) using universal influenza primers according to the Hoffman approach (6). PCR products were cloned into pJEM plasmids and sequenced using m13 primers. Sequencing was carried out in a 16-capillary AB3130xl genetic analyzer (Hitachi Applied Biosystems) with a BigDye Terminator cycle sequencing kit version 3.1 (ABI, Foster City, CA, USA). Raw data were processed with the use of Sequencher version 5 (GeneCodes Corp.) and BioEdit version 7.2.5 for sequence assembling and alignment. The size of each segment of the virus is shown in Table 1.

**TABLE 1:** Genome characteristics of strain A/swan/Mangystau/3/2006

Gene/segment	Size (nucleotides)	GC content (%)	Strain with closest relative sequence	Identity at nucleotide level (%)	GenBank accession no.
PB2	2,341	44.59	A/mute swan/Aktau/1460/2006 (H5N1)	99.83	MK779137
PB1	2,298	44.16	A/mute swan/Aktau/1460/2006 (H5N1)	99.91	MK779136
PA	2,233	43.98	A/mute swan/Aktau/1460/2006 (H5N1)	99.64	MK779135
HA^*a*^	1,769	41.66	A/mute swan/Aktau/1460/2006 (H5N1)	99.89	FJ436942
NP	1,565	47.47	A/greater white-fronted goose/Netherlands/2/2007 (H6N8)	99.30	MK779134
NA^*a*^	1,409	43.50	A/mute swan/Aktau/1460/2006 (H5N1)	99.93	FJ436943
M	1,023	47.50	A/Anas platyrhynchos/Slovenia/359/06 (H5N1)	99.12	MK779133
NS^*a*^	849	42.40	A/mute swan/Aktau/1460/2006 (H5N1)	99.88	JF262041

a Sequences of the HA, NA, and NS segments (GenBank accession numbers FJ436942.1, FJ436943.1, and JF262041.1, respectively) have already been published.

**Fig. 1:**
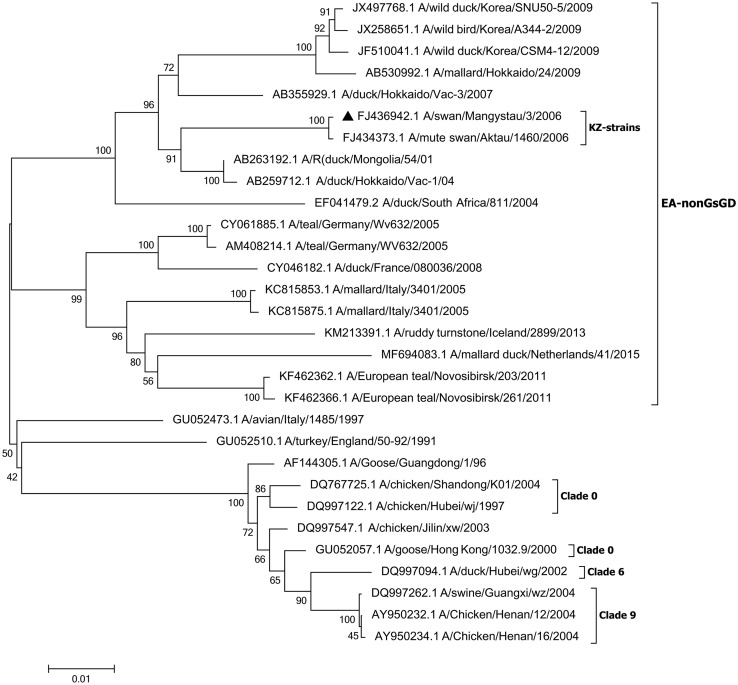
Phylogenetic tree for the HA gene of the strain A/swan/Mangystau/3/2006 (H5N1). Phylogeny of the HA gene was inferred using the maximum likelihood method with 1,000 bootstrap replicates in MEGA version 6.06. The Kimura 2-parameter substitution model was selected with the assumption of a gamma distribution with invariant rates among sites (8). The location of the sequence reported here is indicated with a triangle.

Phylogenetic analyses (Fig. 1) suggested that the HA gene segment of this SW/3/2006 H5N1 virus belonged to the Eurasian lineage, EA-nonGsGD clade, which does not have the main factor determining the virulence of HPAI H5N1 viruses—a polybasic HA cleavage site. Nevertheless, the intravenous pathogenicity index (N) was 2.34, and it allowed the strain to be considered highly pathogenic (7).

### Data availability.

The complete genome sequence of strain A/swan/Mangystau/3/2006 (H5N1) has been deposited in GenBank under the accession numbers FJ436942, FJ436943, JF262041, and MK779133 to MK779137.
